# Community Treatment Orders and Supported Decision-Making

**DOI:** 10.3389/fpsyt.2019.00414

**Published:** 2019-06-11

**Authors:** Lisa Brophy, Renata Kokanovic, Jacinthe Flore, Bernadette McSherry, Helen Herrman

**Affiliations:** ^1^School of Allied Health, Human Services and Sport, La Trobe University; ^2^Centre for Mental Health, Melbourne School of Population and Global Health, The University of Melbourne, Melbourne, VIC, Australia; ^3^Mind Australia, Heidelberg, VIC, Australia; ^4^Social and Global Studies Centre, RMIT University, Melbourne, VIC, Australia; ^5^Melbourne Social Equity Institute and Melbourne Law School, The University of Melbourne, Parkville, VIC, Australia; ^6^Orygen, The National Centre of Excellence in Youth Mental Health, Parkville, VIC, Australia; ^7^Centre for Youth Mental Health, The University of Melbourne, Parkville, VIC, Australia

**Keywords:** community treatment orders, supported decision-making, autonomy, coercion, psychiatry

## Abstract

This paper presents findings from an interdisciplinary project undertaken in Victoria, Australia, investigating the barriers and facilitators to supported decision-making (SDM) for people living with diagnoses including schizophrenia, psychosis, bipolar disorder, and severe depression; family members supporting them; and mental health practitioners, including psychiatrists. We considered how SDM can be used to align Australian laws and practice with international human rights obligations. The project examined the experiences, views, and preferences of consumers of mental health services, including people with experiences of being on Community Treatment Orders (CTOs), in relation to enabling SDM in mental health service delivery. It also examined the perspectives of informal family members or carers and mental health practitioners. Victoria currently has high rates of use of CTOs, and the emphasis on SDM in the Mental Health Act, 2014, is proposed as one method for reducing coercion within the mental health system and working towards more recovery-oriented practice. Our findings cautiously suggest that SDM may contribute to reducing the use of CTOs, encouraging less use of coercive practices, and improving the experience of people who are subject to these orders, through greater respect for their views and preferences. Nonetheless, the participants in our study expressed an often ambivalent stance towards CTOs. In particular, the emphasis on medication as the primary treatment option and the limited communication about distressing side effects, alongside lack of choice of medication, was a primary source of concern. Fears, particularly among staff, about the risk of harm to self and others, and stigma attached to complex mental health conditions experienced by consumers and their families, represent important overarching concerns in the implementation of CTOs. Supporting the decision-making of people on CTOs, respecting their views and preferences about treatment, and moving towards reducing the use of CTOs require system-wide transformation and a significant shift in values and practice across mental health service delivery.

## Introduction

This paper introduces an interdisciplinary project undertaken in Victoria, Australia, to investigate how supported decision-making (SDM) with people who reported diagnoses including schizophrenia, psychosis, bipolar disorder, and severe depression (hereafter consumers) can be used to align Australian laws and practice with international human rights obligations. It examines the experiences, views, and preferences of consumers, family members supporting them, and mental health practitioners (MHPs), including psychiatrists, in relation to enabling “supported” (rather than *shared* or *substituted*) decision-making about care and treatment in mental health service delivery. Victoria currently has high rates of use of Community Treatment Orders (CTOs) which are governed by the *Mental Health Act 2014* (Vic). One of the key objectives of this legislation (set out in section 10) is to support people who are subject to compulsory treatment to make their own decisions about their assessment, treatment, and recovery. However, just how SDM will be used in the context of CTOs is unknown. This paper examines the experiences and views of participants about the relevance of SDM for the reduction of CTO use in Victoria. It focuses particularly on findings from qualitative interviews with 30 MHPs, including community mental health support staff, nursing, allied health practitioners, and psychiatrists, 8 of the 29 consumer interviews, and 10 of 29 family supporter interviews in which the challenge of SDM and CTOs are directly discussed.

## Background

Mental health laws in many jurisdictions around the world enable others to make decisions for people experiencing severe mental health problems, often because of pre-conceived notions about their decision-making abilities. SDM refers to the process of providing support to consumers to ensure that their views and preferences are respected on an equal basis with all others in the community (1). Hence, SDM gives expression to the wishes and preferences of consumers and is contrasted with substitute decision-making, when other people have power to make decisions for consumers, regardless of their wishes. SDM regimes may help ensure mental health laws in Australian states and territories are compliant with the United Nations Convention on the Rights of Persons with Disabilities (CRPD) (2), which requires States to “take appropriate measures to provide access by persons with disabilities to the support they may require in exercising their legal capacity” [Article 12] (3).

The shift to SDM coincides with the increased influence of the recovery approach to mental health practice, policy, and law (2). However, the emphasis on the fundamental themes of hope, social inclusion, and empowerment in the recovery approach (3) appears to contrast with the ongoing and increasing use of involuntary outpatient treatment in Australia through Community Treatment Orders (CTOs) (4). CTOs are controversial in many respects, including concerns about their effectiveness and impact (5). However, they remain entrenched in mental health service delivery.

Despite the CRPD and the stated intention of many governments to reduce the use of coercive interventions, there is a continuing belief that mental health laws need to continue to incorporate substitute decision-making (5). For example, the *Mental Health Act 2014* (Vic) refers to the need for consumers to “be supported to make, or participate in” treatment decisions [section 11(1)(c)] but enables a tribunal to make treatment decisions for consumers, even against their wishes, providing certain legal criteria are met.

There are currently debates regarding the relevance of SDM in jurisdictions that continue to enable substitute decision-making. It has been argued that Article 12 of the CRPD “imposes an obligation on States Parties to eliminate substituted decision-making regimes in their entirety, recognize the diversity of ability to make decisions, and provide a continuum of support to ensure legal capacity”(6). However, Browning et al. (7) describe SDM as a process of supporting a person with decision-making; a system that affords legal status; and a means of bringing a person’s will and preference to the center of any substituted decision-making process (7). Callaghan and Ryan (2) suggest that “a true supported decision-making model will require that all decisions are guided by a patient’s will and preferences—even where decision-making is made *via* a substitute decision maker” (p. 617) (2).

CTOs are controversial in several respects. They operate overall in a way that appears to contradict the shift to recovery-oriented practice and the expectations of Article 12 of the CRPD. They lead to a restriction of human rights including the rights to liberty and physical and mental integrity sometimes over many years, and evidence about their effectiveness remains weak despite randomized control trials (5, 8, 9). Corring et al. (10, 11) have undertaken reviews of qualitative studies on the experience of people on CTOs and suggest that there are common themes among people on CTOs and clinicians (10, 11). They may, for example, see CTOs as helpful but remain concerned about the ethical and human rights implications of their use. However, MHPs tend to see more benefits to the use of CTOs than consumers (10). There is concern about the potential for CTOs to be overused and for those on them to experience coercion and disempowerment. CTOs are also considered “deskilling” and forming a substitute for more innovative, well-resourced and intensive services (12). Yet, informal family supporters have tended to support the use of CTOs and have identified that these orders can assist them (13). The most recent Cochrane review of the evidence for compulsory community and involuntary outpatient treatment for people with severe mental disorders concluded that despite three randomized control trials there was still no evidence that CTOs were effective in reducing clinical outcomes such as hospital readmission and quality of life (8). Large cohort studies in Australia have tended towards more positive findings about clinical outcomes (14, 15), but it is generally concluded that this evidence is not strong (16). Rugkåsa et al. (17) have suggested that on the basis of the evidence available, the common use of CTOs should be reconsidered (17).

## CTO Use in Victoria

This paper explores the relevance of SDM to an estimated 5,000 people who are forced to comply with CTOs in Victoria (18). CTOs were introduced in Victoria under the Mental Health Act 1986, and they have become increasingly commonplace, especially since the mid-1990s when there was a surge in their use as Victoria’s long stay psychiatric hospitals closed. Victoria has the highest rate of CTOs with 98.8 per 100,000 population compared, for example, to 30.2 per 100,000 population in Tasmania and 46.4 per 100,000 in New South Wales (18). In Victoria, CTOs provide the power to return a person to hospital if they do not adhere to treatment or are no longer considered to be able to be treated in the community. The process of recalling people to hospital may involve emergency services, including police (19). Reform of mental health legislation in Victoria has been driven by a range of concerns including the apparent overuse of CTO, concerns about their effectiveness, and human rights issues (13). The reform also intended to promote the Act’s role in supporting recovery and improve the participation of people receiving mental health treatment and care in decision-making (13). The Act introduced more effective and accessible mechanisms to oversee treatment and care and included provisions for enabling improved responsiveness to the needs of families and supporters. The Act is based on principles that include a presumption of capacity and emphasis on SDM (20). Mechanisms to enable SDM were included in the Act, such as advance statements, nominated persons, and a second opinion scheme. These mechanisms enable consumers to express their views and preferences through, for example, recording these in advance or having someone they trust assist them at times when they are less able or unable to do this themselves. Along with several other reforms, Victoria now has a Mental Health Complaints Commissioner, and people on compulsory orders have improved access to advocacy through an independent mental health advocacy scheme. The Act also attempts to promote improved participation in decision-making by supporters.

## The “Supported Decision-Making” Project

This interdisciplinary project involved a collaboration with five peak Victorian mental health service providers and interdisciplinary academics with backgrounds in sociology, psychiatry, law, social work, and population health. The study aimed to explore the barriers and facilitators to SDM in an Australian context. The study included the perspectives of consumers and family members who support them to understand their experiences and seek their views on and preferences for supported decision-making. MHPs, including psychiatrists, have also been interviewed for their perspectives on the provision of treatment and care and SDM (21–23).

### Ethics

The project was approved by the Monash University Human Research Ethics Committee (CF13/2980-2013001607).

### Methods

As described in detail by Kokanović et al. (22) and Knight et al. (23), the project conducted narrative interviews about experiences of SDM with a total of 92 participants during 2014 and 2015 (although two subsequently withdrew) (21, 23). This paper specifically focuses on the experience of participants in these original interviews who had direct experience of CTOs. Twenty-nine participants were consumers who reported diagnoses including schizophrenia, psychosis, bipolar disorder, and severe depression, and eight of those participants had experience of being on CTOs. Thirty interviews with family supporters were also conducted; 10 had direct experience of a family member being on CTOs. All interviews were either video or audio recorded, and the consumer and family supporter interviews contributed to an online resource (http://research.healthtalkaustralia.org/supported-decision-making/overview). Ten psychiatrists (nine from Melbourne and one from a regional area) and 20 MHPs were interviewed. Mental health services in Victoria are separated into non-clinical [non-governmental organisation (NGO) or Mental Health Community Support Service (MHCSS)] and clinical services. The MHPs interviewed comprised staff from both services, including peer support workers, social workers, nurses, occupational therapists, and community mental health support service practitioners (21, 22). All these participants had experience working with consumers and their families on CTOs.

Recruitment of consumers and family supporters occurred through posters, staff contact, and email networks in community mental health organizations. Recruitment of MHPs and psychiatrists included information distributed at sector events and through professional associations (21, 22). Advertisements and posters included researchers’ contact details, and potential participants were invited to initiate contact. All interviewers were academics employed by the universities involved in the study, and there were no previous relationships or contact between interviewers and interviewees. LB undertook five of the interviews with consumers and all the interviews with MHPs. RK undertook the interviews with psychiatrists. The rest were conducted by the research team. There were no direct relationships between the consumer interviewees and MHPs interviewed. They were representatives of their professions. Further, there was no direct relationship between the consumer participants and the family supporters interviewed.

MHPs and psychiatrists were asked about their experiences in the implementation of CTOs, their understanding of the role of CTOs in service delivery, and how CTOs impacted their relationships with consumers, their families, and other informal supporters. Consumers and family supporters who had experience of CTOs were asked about their, or their family members’, experiences and how being on a CTO impacted them and their families in the context of a broader discussion about living with a diagnosis of mental illness, their experiences of care, SDM and recovery [see Ref. (22) for more details].

The data were analyzed thematically across all participants after the interviews had been transcribed, returned to participants for review, and imported into NVivo 10 software for qualitative data management (22). The primary analysis involved the development of coding frameworks by the research team. Using an experience-centered approach, the research team members were involved in cross-checking the analysis and developing the coding frameworks (24) [see Refs. (22) and (23)], and findings have been published elsewhere (19, 21). Common and divergent themes among participant groups were identified in the secondary analysis of the 48 relevant interviews for this paper that explored study participants’ accounts and interpretations of their experiences with CTOs and SDM. The inductive approach to coding involved several sessions of reading the transcripts and identifying themes specifically referring to CTOs. LB initially formulated a coding framework that was discussed with the rest of the research team as it developed. This was complemented by analysis undertaken to produce the two digital resources that both include sections on CTOs and SDM (http://research.healthtalkaustralia.org/supported-decision-making/overview).

## Findings

### Perspectives on Supported Decision-Making and CTOs

#### Mental Health Practitioner and Psychiatrist Perspectives

In this section, we provide an analysis of findings related to the implementation of CTOs from the interviews with 20 MHPs and 10 psychiatrists who had direct experience of implementing CTOs. They describe navigating a very complex system. CTOs appear to be more than an order that impacts the named individual. The effects are disseminated to families, the way the mental health system operates, and the relationship the person has with the service and providers. This complexity also relates to differences in power, with psychiatrists being the most powerful decision makers while CMHSS workers appear to have the least. There are also different opinions about whether CTOs are helpful and whether they could be doing harm due to the impact of coercion and the cost they pose to relationships between consumers and service providers. SDM was seen as relevant to these issues because of its potential to increase opportunities for greater respect for autonomy, human rights, and choice and control (22) and because it provided a challenge to the other imperatives driving the use and reliance on CTOs. Finally, as we discuss below, some MHPs and psychiatrists were able to identify signs of change in the implementation of CTOs in the current context.

##### Power and Influence

The implementation of CTOs is particularly challenging for recovery-oriented practitioners in CMHSS who are working in partnership with clinical services. CMHSS workers, as non-clinical service providers who do not hold powers under the Act, described readily embracing recovery-oriented practice as their guiding framework. Hence, they expressed an acute awareness of the tensions between coercive practices such as CTOs and recovery. CMHSS participants described their sense of paternalism and disempowerment when working with staff from clinical services leading to feeling marginalized within a hierarchical system, even though the relationships they have developed with consumers could provide an important contribution to influencing decision-making about treatment and CTOs. For example,

Yeah, I got told not to coach the client [by a psychiatrist], not to answer questions, and then I said, “If she looks at me and wants me to respond, is it okay?” I felt like I just had to keep my mouth shut … [about what I knew about the person] (Cassie, CMHSS worker, Occupational Therapist).

This quote exemplifies the difficulties discussed by participants working in CMHSS. While CMHSS provide the most intensive and direct individual support to consumers, they hold very little power in relation to contributing to decision-making about matters such as the need for a CTO.

However, psychiatrists also recognized their own difficulties as the more powerful decision makers in the process of implementing CTOs. They felt they were the ones most likely to be blamed if any harm occurred to the person or others involved if they did not order or continue a CTO. This led them to rely on CTOs even though this potentially conflicted with other practice principles, such as respect for autonomy. It also had the potential to be “deskilling” for psychiatrists and other MHPs due to relying on coercion to achieve medication compliance, rather than exploring other ways to encourage people to take their medication:

I think many doctors felt that they had to put people on CTOs because if they didn’t and then something bad happened then they would get blamed, and also I think that because CTOs were available I think a lot of doctors haven’t developed the skills in trying to … collaborate with people and,… you know, persuade people that medication in particular was—was a valuable thing for them (Sam, Psychiatrist).

##### Safety at a Cost to Relationships and Autonomy

Clinical staff commonly referred to people being “safe” when they are on CTO and CTOs helping them meet their duty of care obligations, but they also most commonly described the experience of being on CTOs in negative terms. The use of CTOs appeared to sometimes interfere with developing a trusting relationship due to power differentials being highlighted, and CTOs also formed a substitute for other ways of working that might be riskier such as enabling consumers to make their own decisions about their medication and other aspects of their treatment and care. For example,

People hate being on a CTO. It’s, it’s like their, their autonomy is taken away. And I still think we don’t [um], we don’t properly ascertain what are the real problems that are going to happen to the person. I mean, I still think we’re being very paternalistic. We’re not letting people make mistakes or find out for themselves (Rufus, Psychiatrist).

These views were not universal among the respondents, with accounts of neutral to positive attitudes towards CTOs. Some respondents were in favor of using substitute decision-making because it was in the consumer’s best interests. These participants identified a CTO as helping them to meet their duty of care and preventing harm to the consumer or others. In some situations, they were not concerned about the coercive aspects of CTOs because the consumer did not seem to object to being on a CTO. For example,

She [the consumer] honestly didn’t care about the order. She [the consumer] didn’t really understand it; it was just a piece of paper to her (Gwen, Nurse).

##### Enforcing Medication

All the MHPs and psychiatrists saw the primary role of CTOs as enforcing compliance with medication. They were aware that this gave them control over the type of medication and dosage prescribed and led to consumers having reduced opportunity to negotiate these decisions:

We have a lot of people that sort of want to reduce the amount [of medication] that they take. Some people do want to increase it as well but I guess it’s that you know people feeling bombed out and got no, no energy and things like that and they’re like how can I, how can I get out of this? Usually if they’re on a Community Treatment Order that’s a little bit trickier (Siobhan, CMHSS worker).

Several MHPs were not convinced that the benefits that medication might offer was enough to justify the coercion. One peer support worker thought he could never recommend giving a person a depot or intramuscular injection of medication (which is commonly used in Victoria when people are on CTOs) against their will, even though he thought CTOs were necessary sometimes: *“I know how bloody intrusive, and I’m not saying they’re not a good thing, and like I said before, sometimes they’re necessary, but having been there myself I just, I just—that’s where my empathy comes in”* (Seamus, Peer Support Worker).

##### Continuity of Care and Access to Scarce Services

Most participants identified CTOs as having an important role in the current system of providing mental health care. CTOs were seen to have functions other than medication compliance, such as facilitating people to stay out of hospital by guaranteeing that they would receive timely follow-up. Several participants identified challenges to reducing the use of CTOs, because of the role the orders appear to have in enabling improved continuity of care and ensuring access to service delivery. One psychiatrist shared their experience on an inpatient unit as follows:

The consultant [psychiatrist] had very strong, you know, views … everyone went out [of hospital] on a treatment order. Everyone … pretty much … who came in with a psychotic type illness. In, out on a treatment order because that way they’d get community follow up (Joseph, Psychiatrist).

Some participants commented that access to services can sometimes *require* a CTO rather than prevent one:

They say, oh well if they weren’t on an order, MST [the Mobile support and treatment team] probably wouldn’t … keep them on their books because they only take the most severe people. But that’s like totally putting the cart before the horse, you know … Like surely the whole point of MST is to get people to a point where they can be self-determining and autonomous and make their own decisions. It’s really—you know, why, why, why should you need to be on an order to get a service, that doesn’t make any sense (Sam, Psychiatrist).

There were also challenges to reducing the use of CTOs and engaging in more SDM related to continuing support for families:

I think families feel safer that their loved ones are on orders … in the sense that they know it’s going to access them to mental health services (Sophia, Social Worker).

##### Ambivalence—CTOs Fix Some Problems and Create New Ones

Most participants expressed ambivalence about the effectiveness of CTOs. They were often seen as a “blunt instrument” used to address other systemic problems, such as access to service delivery (as described above), addressing the fears of organizations primarily concerned about preventing the risk of harm to consumers and others, and making people accept unpleasant and sometimes distressing treatment. However, while CTOs might be addressing some problems, they were potentially creating others, such as the possibility that a CTO might have a negative impact on the person’s future engagement with services:

It’s a lose-lose situation no matter what you do. You keep them on the CTO and it’s quite—it’s, it’s quite sad. But that’s the sad part is sometimes that you take away someone’s identity and—because that would be, I think, for her self-esteem personally that would be a serious loss. The fact that you’re forced to do something that’s against your, your beliefs and you have—and no matter what you do, if you stop taking the medicine then you’ll be sent to hospital and you’ll be taking it anyway and that’s, that’s quite sad or sad to me (Mavis, CMHSS worker).

On the other hand, many participants thought CTOs could be justified with some people because of the severity of their symptoms, even if the consumers on CTOs found this very distressing:

I think regrettably the people who probably hate the CTOs the most are people who are more likely to get really severely unwell (Geraldine, Psychiatrist).

One participant thought the Act and its safeguards enabled human rights protections for consumers on CTOs that sometimes were not available to others who were pressured to cooperate:

Because they don’t get any of the protections under the provisions of the Mental Health Act. So I be very kind of clear with case managers and, you know, and when, when the team is like, “Look, you know, if we’re going to go down, if we actually are going to use such a treatment and use treatment pressure stuff, you need to, you know, use the legislation and make sure that it’s done appropriately,” and…, they have the protections and they can appeal it (John, Psychiatrist).

##### SDM and CTOs

As discussed above, many MHPs and psychiatrists experienced pressure to use CTOs despite several also feeling ambivalent about them. A fundamental problem commonly identified was the negative impact of CTOs on the autonomy and empowerment of consumers. SDM appeared to have the potential to increase opportunities for greater respect for autonomy, because it facilitates consumers being able to express their views and preferences about treatment and care, as this participant describes:

Absolutely. I think through supported decision making you’re going to give people ownership of, of not only themselves and, and their illness and their treatment but that sort of responsibility I think will create investment in taking the medicine. I mean if you’re twisting people’s arms with CTOs or anything like CTOs or like forensic orders or anything like that, you’re just twisting people’s arms and then at some point they’re going to come off those orders and … I think if you support their decision-making, perhaps at least then they’ll invest themselves in it and then they will do that or maybe they’ll do some of it (Mavis, CMHSS worker).

Active engagement in SDM with people on CTOs included informing them about their legal rights and reassuring them about the powers that a CTO enables:

I think currently from my own experience I’ve had a couple of people that are really confused about the CTOs in particular. A fear that what they’re hearing from clinicians or, or clinical staff members is not in fact what they—well, the—essentially the truth (Stuart, CMHSS worker).

MHPs also identified other opportunities for people to “get control back” while on a CTO. This may not be possible in relation to taking medication and complying with clinical services, but MHPs identified opportunities for hearing more about consumers’ views and preferences and supporting these in relation to, for example housing, employment, and finances:

You can still support them [people on CTOs] to make decisions regarding … whether it be they want to look at employment or education opportunities; there’s still a huge other scope. You know, whether it’s engaging in kind of a leisure activity or—there’s still a huge scope to be able to help them to make supported decisions in other areas (Cassie, Occupational Therapist).

Another example provided was for services themselves to adapt and enable people to have more choice and control in their treatment. For example, medication supervision:

When you’ve got people [on CTO] saying “please don’t come to my door at six o’clock at night because it doesn’t suit me. That’s not when I take my medication. I take it later on in the night. Now can you come back at 8:30?” (Karen, Nurse).

This participant then related this seemingly common scenario to how difficult it can be for someone to regain control over their lives and have their preferences respected. In this scenario, family relationships were important, and SDM relied on family members supporting a shift in the balance of power:

Tensions with family, you know; why aren’t you taking the medication? Why are you arguing, arguing with these, these experts that come to the door? Like it creates a whole knock on effect of things … and so, so those little things, those little examples which are really a big deal, I don’t know that we’re really thinking about those things … around supported decision-making. So they’re not captured in the advance statement, but they don’t need to be. It’s just we as a service, how do we think about those things on a day to day basis? How can we really support people to make their own decisions? (Karen, Nurse).

Attempts to try to work towards SDM with people on CTOs were described by MHPs as requiring time for conversations and information sharing with consumers, following through with agreed assistance and acting on consumers’ views and preferences:

Which means discussion on the medication and its side effects. And if they have a view on one versus the other, then—then do that (Stephan, Psychiatrist).

##### Signs of Change

Participants commented that there were “signs of change” since the Mental Health Act, 2014 had come into effect. In many cases, these signs confirmed MHPs’ and psychiatrists’ perspectives that SDM was relevant to the implementation of CTOs, as evident from quote below:

I think we’re getting a little bit better. I think people now know that they have an avenue through the Mental Health Complaints Commissioner that they have an avenue to talk, they have an avenue to speak. I think people overall … are finding that they’re [CTOs] are shorter in duration. That … they’re being given a chance … to express what they want, what they don’t want. And I think also too that I am … as a … senior clinician who has been around for a while now that those more conservative consultants [psychiatrists] are having to actually think about the future. They’re actually thinking more about treatment goals and recovery and that it’s not a—a mental illness doesn’t actually mean you’re in a constant state of … illness and you’re not in a constant state of incapacity (Sophia, Social Worker).

Participants identified SDM as having significant potential to improve the implementation of CTOs through encouraging less reliance on these orders, as well as enabling consumers on CTOs to express their views and preferences more. However, for some participants, this change had not come fast enough or may not be as effective as they hoped. They identified how incongruous several mechanisms to enable SDM, such as advance statements, were with people being on a CTO and how a lack of advocacy contributed to the problem:

if … we are going to treat this person involuntarily … how are we going to get them to do an advanced statement for that?… I think frontline staff do navigate that quite well. Because they’re the ones that have to go and give people medication against their will and all of that kind of stuff. I just think some like ways of recognising that … they could get an advocate (Clementine, Nurse).

#### Consumer Perspectives

The following section provides findings from interviews with 8 of the 30 consumer participants who had direct experience of CTOs. It describes their varied experiences of being placed and living on a CTO and their role in decision-making. For some the impact was wholly negative, while others saw some benefits. Many could identify a rationale for why they were put on a CTO, even if they did not agree with this decision. Others reported a lack of information and feelings of powerlessness that pervaded the whole experience. Being forced to have medication was an important theme, in both understanding the purpose of CTOs and in relation to distressing experiences attributed to being on a CTO.

##### Views on Why CTOs Were Implemented and Their Impact

Most consumers thought CTOs were made because they were “non-compliant” or identified by treatment providers as someone who was potentially at risk and might harm themselves or others if they did not take medication as prescribed. Events from the past and when the person was unwell were usually used to explain the CTO to participants, but many felt that there was not enough recognition that things had changed. However, participants also described benefits from the support and treatment obtained while on a CTO including medication and psychosocial support:

for me, I, I think it was necessary … because I could have, I could have gone on you know longer until … you know some circumstances, because I was, I was doing some silly things that could have got me killed (Cheryl).

##### Transparency, Information, and Decision-Making

Most participants described not getting much information about CTOs and why they were placed on one. However, one participant described the CTO as a (welcome) way to get out of hospital, and because it was consistent with his own priorities, he did not object or even seek more information at the time. This is a continued reflection on how CTO decision-making is in the hands of the service providers and—at least for this group of consumers—there was minimal evidence that they had been involved in the decision as the following quote indicates:

There was never a conversation about CTO, being put on a CTO. It was only the last conversation with my psychiatrist from the psych ward when she last came to see me before I was being discharged, that’s when she told me. She goes, “You’ll be put on a CTO and which means you have to comply with your medication and you have to go down, have your injection.” Like she just described it to me and I said, “All right, yeah. I’ll do that, yeah.” But at that point in time after you’ve been in the psych ward for six weeks, you’ll agree with anything to, you know, just leave for a bit (Amrick).

Some of the experiences of disempowerment people feel on CTOs was related to this lack of information and fear of the mental health system:

I wasn’t told nothing about that so I didn’t know what was going to happen about the treatment order yeah. Oh it was nerve-wracking … you kind of felt didn’t know, didn’t know what was going to happen so you had to kind of be patient and wait for it to see what was going to happen (Lily).

Participants identified CTOs and this lack of information as increasing their experience of stigma. For example,

I read it (the order) and it was after when she left I read it and then I, and then I thought oh my goodness they’ve put me on something, I mean I felt like I was a criminal (Yolanda).

##### Being Forced to Take Medication and Endure Side Effects

Medication side effects, their impact, and not feeling heard about them, was a key theme in the consumer interviews. Many thought the CTO was, in some ways, forcing them to tolerate side effects. They considered this an injustice because of how unpleasant the side effects were and the significant lack of autonomy that not having choice about mediation represented. Thus, descriptions of feeling disempowered and lacking choice and control often featured in discussions about side effects as follows:

Because you know a few of the medications I’d had before that, I’d had you know really bad side effects from and I just was like no, I’m not taking this … the side effects are just making me miserable (Cheryl).

##### Disempowerment

Most consumers referred to their experiences on CTOs as disempowering and stigmatizing, even if it had not been particularly distressing. Participants also described the loss of choice and control about treatment and care that comes with being on a CTO. This was also linked to the participants’ dissatisfaction with treatment they were receiving, the lack of trust people had in them, and not being heard:

I feel I have no choice in the matter really. That’s the way I feel. Like either it’s going to be taking the—taking the depot or feeling sick. So that’s the only two choices I have and even if I do try to tell the doctor, you know, can we change medications, I don’t think the point’s getting across to them. So I don’t—I, I can only take the medication, that’s it (Amrick).

##### Consumer Perspectives on SDM and Self-Advocacy

Participants talked about decision-making in more general terms without specific reference to SDM. However, they had all been involved in substitute decision-making, and their accounts indicated links to SDM through references to strategies they employed for their views and preferences to be heard. They recalled relying on self-advocacy when they tried to improve their situation, perhaps through a change of medication, a reduction in the dosage, or asking to be discharged from the CTO. Some attempted to self-advocate through conversations with their treating team and others through getting independent access to information. Some participants described seeking help from other professionals or the Mental Health Review Board (now tribunal). No participant referred to ever having had an independent advocate. For at least one participant their self-advocacy had to extend to convincing family members that they could be discharged from the CTO. A few consumers also acknowledged the disparity in approaches to how CTOs were administered, with some articulating the benefits of approaches consistent with SDM such as information-sharing and inclusion in the process and, in turn, increased empowerment.

I just followed the psychiatrist until I started lactating. And that’s when I realised that I needed to sort of stand up for myself a bit better. Because I was certainly well and I read the brochure they had in the waiting room about … being on a Community Treatment Order. And I read through the criteria and I thought I definitely—you know I’m definitely well so I don’t belong on this CTO. So it’s time that I, you know, stand up and just say to them, look you know you’re keeping me on this treatment order and I’m well but I’m also compliant, I’d been compliant for—I think it was 10 months (Alejandra).

#### Family Supporter Perspectives

In this section, the experiences of family members who were supporting their relative on a CTO are described. As with other participants, their perspectives are mixed. Many identified CTOs as helpful especially through influencing their family member to take medication. However, they also expressed concerns about getting help and support and not always being sufficiently involved in the processes around CTOs. There were concerns among family supporters that CTOs may be used too readily without exploring other options to understand why their family member was not taking their medication. Some hoped that the new Act would encourage improved communication, but others were also concerned that the new Act might lead to more people being discharged from their CTOs, leading to risks of relapsing or disengaging from services. While some supported their family member to gain more autonomy and respect, they appeared to be fearful that this came at a price for them in potentially having to deal with negative consequences such as their family member becoming unwell or needing to go to hospital. Themes that emerged from the family supporter interviews suggested that there were problems carers identified in the implementation of CTOs. These included that the orders were sometimes just part of the routine rather than a well-considered intervention, could be perceived as a punishment, and were not always the best solution.

##### Problems With the Implementation of CTOs

One family supporter expressed concern that CTOs were often not implemented in ways that met the needs of their families over the long term, suggesting they were either inflexible and not available as a short-term option, or only used for crisis management. Others talked about clinicians relying on CTOs and this just being a routine option without much thought being given to the purpose of the order:

You know, clinicians and clinical staff just fall into the trap of, “I’ve seen this before, we better do this.”Rather than taking each situation on its [um], you know, uniqueness (Nicole, Sister).

Three family supporters discussed how CTOs can be perceived as a punishment:

I did say to my son, “If you wanted to go to the legal aid I’m there to support you.”And he said, “Why?If I get sick so they, they would treat me bad and they would extend the CTO.” He was scared (Tatiana, Mother).

##### Reducing Worry and Uncertainty

Most family supporters noted that CTOs sometimes helped alleviate worry and uncertainty while the person they cared for was on the order. Sometimes this related to serious concerns about harm to the person or others, but mostly it was about how CTOs encouraged the person to take medication.

Lots of times, he’s been on everything, yes lots of Community Treatment Orders yes, and he does stick by them when he’s on them. Which is good, because you know that he’s going to take his medication, and you’re going to have a bit of relief for a while. So, but then they don’t last forever, and then he goes off them (Wendy, Mother).

Family supporters often identified the CTO as helping to prevent the person relapsing, and this contributed to them not having to worry so much. A few family supporters expressed concern about the wellbeing and recovery prospects of the person they were supporting, should they experience a relapse of symptoms if they were taken off the CTO:

Each time he has an episode, I’m sure it’s worse for his brain (Penny, Mother).

##### CTOs Are Not Always the Solution

Even in the context of reduced worry, most family supporters expressed frustration about CTOs often not being the best solution to the problems faced by the person they cared for. They described how CTOs and forced medication seem to be the only response from service providers available to people not taking their medication, and having a crisis or an admission to the inpatient unit. They described how often the situation can be much more complex than the person not taking medication. Family supporters talked about the lack of support for issues such as addiction to substances and the need for other therapeutic interventions:

Every time discharged on a CTO but unfortunately it hasn’t been any treatment except every fortnight he have to go and see the case manager for his depot and the psychiatrist every three/four months, sometimes more than four months (Tatiana, Mother).

Some family supporters expressed concerns that MHPs could rely on a CTO to encourage their family member to take their medication, rather than taking more time to understand why the person was not taking medications prescribed.

Like, I don’t think that non-compliance should necessarily equate to, “You need to go on a Community Treatment Order”. I think, you know, there are so many factors that go into an episode and, you know, people are just a lot more complex than drawing a straight line between those two things. So in my brother’s case, when he’s had, you know, 15 years of good compliance, became unwell and then it was suggested that he go on a Community Treatment Order. We had to fight really hard for that to not happen. (Nicole, Sister)

##### Lack of Involvement in Decision-Making

Family supporters commented that the processes around CTOs, particularly under the previous Act, had made their involvement difficult. Hence, their concerns and their role in providing ongoing care were not necessarily taken into consideration. Some described the difficulties of providing support when their family member was taken off a CTO. This particularly impacted on their ability to access support from the treating team to, in turn, support their family member not to become unwell and return to the hospital.

But quite often carers do get upset that they’re not being heard (Hannah, Stepmother).

##### Opportunities for Supported Decision-Making in Changing Legislation

Supporters also saw opportunities in changes that had been made to legislation. One supporter described the establishment of the new Mental Health Tribunal as “huge.” She was optimistic that it would be easier for supporters to access information and attend CTO hearings:

I’ve got a feeling that it’s meant to be easier for carers to have access to the information, for them to have an opportunity to attend the hearing (mental health tribunal)… I think staff and clinicians need to talk in a way that carers understand, but you know, as a carer it’s good to learn some of the terminology so that, you know, you have more understanding of the system. (Natalia, Mother)

Some supporters were concerned about some of the changes effected by the new mental health legislation. They worried that the reduction of CTO use, in line with the intentions of the principles of the new Act, could lessen their capacity to support their family members to take their medication when they came home from hospital.

By contrast, another supporter agreed with the principle that all people should be able to make decisions involving risk:

I guess life is a risky business and so people need to be able to take their own calculated risks (Raewyn, Sister).

## Discussion

Consistent with previous qualitative research in Australia and elsewhere, service providers, family supporters, and consumers shared many similar perceptions regarding the experience of CTOs, as well as a mixed and often ambivalent stance about CTOs (10, 11, 25, 26). Most consumers described CTOs as wholly negative; both the process of being placed on a CTO and its impact. Others saw some benefits related to getting treatment when they were unwell or being discharged from hospital. Psychiatrists and MHPs were concerned about the disempowering impact of CTOs but continue to see the orders as sometimes necessary to meet their duty of care obligations. Family supporters identified benefits in the use of CTOs, while also expressing concerns, including overemphasis on medication compliance and lack of other therapeutic interventions. Other sources of concern or ambivalence about CTOs related to their potential for overuse and their impact on relationships between services providers, consumers and family supporters. As Light et al. (12) also found, participants appeared concerned that CTOs sometimes act as a substitute or antidote for other practice and systemic problems, even seeing their use as a way to guarantee continuing access to care under conditions of relative scarcity of resources (12).

This study asked consumers to reflect on their experiences on CTOs in relation to supported decision-making. Very few consumers described experiences where they were supported to make decisions about their treatment. It was usually only when side effects or symptoms became unbearable that they were catalyzed into participating in decisions. However, many of the MHPs, psychiatrists, and family supporters appeared hopeful about the potential for mechanisms introduced by the Act to enable positive change in CTO implementation. Many identified positive outcomes for consumers and were already seeing “signs of change.” Nonetheless, there were fears that increased emphasis on SDM would result in reducing the number of people on CTOs, which could lead to consumers being subsequently abandoned by services providers. Davidson et al. (27) have previously observed that an emphasis on increased autonomy may have the unintended consequence of benign neglect when services are not equipped for less reliance on coercive interventions (27). Vine and Judd (28) have also recently described how reduced funding in Victoria for mental health services heightens this risk (28).

There was some divergence in emphasis that may be an important contribution to understanding different stakeholder experiences with CTOs. Consumer concerns regarding experiencing and not being heard about medication side effects, and the degree to which this is central to their experience of CTOs, appear to be important. It suggests the ongoing difficulties the consumers are experiencing when what is so critical to them may not be as significantly appreciated by service providers. Similar to Lawn et al. (29), this was undervalued by some practitioners, suggesting fundamental problems associated with the sometimes pragmatic and instrumental use of CTOs to address systemic problems or deal with practitioners’ own fears (29).

A focus on SDM may assist in aligning Australian mental health legislation with international human rights obligations (30). MHPs and psychiatrists were strongly aware of the potential for the new mechanisms enabled by the Act to facilitate SDM and appeared to have some optimism about the possibilities for SDM. However, the consumers and family supporters appeared to have little information about this. Diverging views may have been because some participants were reflecting on past experiences and were not currently as actively engaged with the mental health system. This also suggests that there is significant room for further progress in changing practice. Trends in the use of CTOs and people’s experience of their use makes a valuable contribution to the question of whether jurisdictions are keeping pace with international expectations about reducing coercive interventions and enabling choice and control. The findings here point to only limited impact so far.

Consumers were more likely to see self-advocacy and personal empowerment as most influential for positive change, rather than mechanisms imposed or enabled by policy or legislation. Family supporters particularly identified improved communication as one of the most important mechanisms to enable their involvement in providing support to their family member. However, the persistent problem of family supporters not feeling heard and lacking opportunities to communicate their perspective was evident (31).

Thus, the findings in relation to this subgroup of participants commenting on CTOs are consistent with our overall project findings (21, 22). Implementation of SDM to achieve positive outcomes for consumers, including reduced reliance on coercion, requires an integration of legal mechanisms, interpersonal skills, consumer empowerment and advocacy, and management and leadership that includes adequate resourcing of community-based services (21, 22).

## Strengths and Limitations

These findings are part of a larger study that did not specifically focus on CTOs (21). Hence, the extracts are taken only from the relevant interviews. There is a relatively small number of participants whose experiences of CTOs could be drawn on for this paper, some of whom were not currently on a CTO. Members of the police, legal practitioners, and tribunal members were not included among those interviewed even though they are also important stakeholders in relation to SDM and CTOs. This suggests the value of extending qualitative research to include broader stakeholder experiences in future.

## Conclusion

There was a general agreement among a diverse range of MHPs and psychiatrists, family supporters, and consumers that SDM has a role in CTOs. Their comments were focused on reducing experiences of coercion and moving towards less reliance on CTOs. The findings also provide some confirmation that current efforts to introduce mechanisms to enable more opportunity for SDM are relevant to people on CTOs, even though participants acknowledged this would require considerable changes in practice to encourage greater focus on respecting the views and preferences of people on CTOs. It appears that the aspiration to give people more choice and control is currently limited in practice. Imbalances of power persist in the service system in Victoria, thus limiting the influence of human rights-based principles. Participants described medication as the main, sometimes only, form of treatment and that clinicians have a low tolerance for “non-compliance.” Consumer perspectives are important in describing and highlighting the long-term disempowering and stigmatizing impact of CTOs and the distress associated with perceived inability to discuss medication side effects. Family supporters were often caught between seeing benefits in CTOs but also concerned about the quality of care. A key issue for them in SDM was improved communication. Consumers appeared to locate opportunities for development of self-advocacy as the most effective way to ensure their views and preferences are heard and respected.

Perceived fear, risk of harm to the consumer or others, and stigma represent important overarching issues in the implementation of CTOs and in enabling SDM. While challenging stigma and respecting the “dignity of risk” was acknowledged by all, these participants still expressed considerable fear about people having more autonomy and the potential consequences. This extended to the potential for consumers to lose access to scarce mental health resources if not on a CTO. The shift to incorporating SDM into the implementation of CTOs requires system-wide transformations and a significant shift in policy, values, and practice in all mental health service delivery contexts. Attention to ensuring that consumers are heard and empowered, that staff have the necessary skills and resources, that mechanisms such as advance statements are fostered, and that management and leadership support system change appear to be essential factors behind attempts to align Australian laws and practice with international human rights obligations (21, 22).

## Ethics Statement

This study was carried out in accordance with the recommendations of the Monash University Ethics committee with written informed consent from all subjects. All subjects gave written informed consent in accordance with the Declaration of Helsinki.

## Author Contributions

LB drafted the manuscript. RK and JF contributed to the data analysis and made substantial contributions to the final manuscript. BM and HH provided expert contribution and comments on later drafts.

## Funding

This project was funded by the Australian Research Council Linkage Projects scheme (LP130100557).^1^

## Conflict of Interest Statement

The authors declare that the research was conducted in the absence of any commercial or financial relationships that could be construed as a potential conflict of interest.

## Footnotes

1 For more details see https://socialequity.unimelb.edu.au/__data/assets/pdf_file/0006/2701626/Kokanovic-et-al-2017-Mental-Health-and-Supported-Decision-Making-ARC-LP-Project-Report.pdf
